# The Bacterial Two-Hybrid System Uncovers the Involvement of Acetylation in Regulating of Lrp Activity in *Salmonella* Typhimurium

**DOI:** 10.3389/fmicb.2016.01864

**Published:** 2016-11-17

**Authors:** Ran Qin, Yu Sang, Jie Ren, Qiufen Zhang, Shuxian Li, Zhongli Cui, Yu-Feng Yao

**Affiliations:** ^1^Key Laboratory of Agricultural Environmental Microbiology, Ministry of Agriculture, College of Life Sciences, Nanjing Agricultural UniversityNanjing, China; ^2^Laboratory of Bacterial Pathogenesis, Department of Microbiology and Immunology, Institutes of Medical Sciences, Shanghai Jiao Tong University School of MedicineShanghai, China; ^3^Department of Laboratory Medicine, Shanghai East Hospital, Tongji University School of MedicineShanghai, China

**Keywords:** lysine acetylation, bacterial two-hybrid, Lrp, DNA-binding, *Salmonella* Typhimurium

## Abstract

N^𝜀^-lysine acetylation is an abundant and important Post-translational modification in bacteria. We used the bacterial two-hybrid system to screen the genome library of the *Salmonella* Typhimurium to identify potential proteins involved in acetyltransferase Pat – or deacetylase CobB-mediated acetylation. Then, the *in vitro* (de)acetylation assays were used to validate the potential targets, such as STM14_1074, NrdF, RhaR. Lrp, a leucine-responsive regulatory protein and global regulator, was shown to interact with Pat. We further demonstrate that Lrp could be acetylated by Pat and deacetylated by NAD^+^-dependent CobB *in vitro*. Specifically, the conserved lysine residue 36 (K36) in helix-turn-helix (HTH) DNA-binding domain of Lrp was acetylated. Acetylation of K36 impaired the function of Lrp through altering the affinity with the target promoter. The mutation of K36 in chromosome mimicking acetylation enhanced the transcriptional level of itself and attenuated the mRNA levels of Lrp-regulated genes including *fimA*, which was confirmed by yeast agglutination assay. These findings demonstrate that the acetylation regulates the DNA-binding activity of Lrp, suggesting that acetylation modification of transcription factors is a conserved regulatory manner to modulate gene expression in bacteria and eukaryotes.

## Introduction

N^𝜀^-lysine (de)acetylation, as a key post-translational modification, plays critical roles in various biological processes, including transcriptional regulation, aging, and metabolism (Grunstein, 1997; Cohen et al., 2004; Lu et al., 2011). Recent mass spectrometry (MS) efforts have revealed that acetylation modification frequently occurs in bacteria. A large amount of acetylated proteins have been identified in bacteria including *Escherichia coli* (Weinert et al., 2013; Zhang et al., 2013; Kuhn et al., 2014; Schilling et al., 2015), *Salmonella* Typhimurium (Wang et al., 2010), *Bacillus subtilis* (Kosono et al., 2015), and *Mycobacterium tuberculosis* (Liu et al., 2014). There are two distinct mechanisms in bacteria to regulate the protein acetylation. The first one is enzymatic, whereby the Gcn5-like acetyltransferase Pat (protein acetyltransferase)/YfiQ transfers the acetyl group from acetyl-CoA (Ac-CoA) to a deprotonated lysine. The other is non-enzymatic, whereby acetyl phosphate (AcP) serves as the acetyl donor to a deprotonated lysine (Hentchel and Escalante-Semerena, 2015; Wolfe, 2016). Nicotinamide adenine dinucleotide (NAD^+^)-dependent CobB (NAD^+^-dependent protein deacylase) has been known as the only lysine deacetylase (KDAC) for a long time (Starai et al., 2002). However, current study uncovers that YcgC, representing a new protein deacetylase family, also exhibits KDAC activity. In addition, YcgC targets a distinct set of substrates from CobB (Tu et al., 2015).

The yeast two-hybrid method was initially developed (Fields and Song, 1989), afterward, the bacterial two-hybrid (BTH) variant was further developed to characterize interactions between two proteins (Karimova et al., 1998). BTH has been widely used to screen for previously unknown protein partners involved in various processes, including antibiotic resistance (Domain et al., 2007), bacterial viability (Handford et al., 2009), spore wall synthesis (Kleinschnitz et al., 2011) and virulence (Klepp et al., 2009). Moreover, it has been shown that BTH is able to detect protein interactions that occur either in the cytosol or in the membrane (Houot et al., 2012). Therefore, in the present study, BTH was employed to identify the interaction proteins with Pat or CobB, so as to explore more unknown acetylation proteins. In contrast, it is hard to identify acetylation membrane proteins by MS since the process of preparation of sample will remove almost all membrane proteins. Using genome libraries of the *S.* Typhimurium strain 14028s, we screened potential targets involved in interaction with Pat or CobB and validated these potential substrates by (de)acetylation assays *in vitro.* Notably, Lrp (leucine-responsive regulatory protein), the leucine-responsive global regulator (GR), has a strong interaction with Pat. GRs are transcription factors that, collectively, play an essential role in bacteria, and coordinate the responses of the thousands of genes in cells to complex environmental changes (Madan Babu et al., 2006). The genes controlled by each GR (its regulon) can specify a variety of disparate functions (Martinez-Antonio and Collado-Vides, 2003; Saxer et al., 2014). The number of regulation targets of Lrp continually increased concomitant with the advance of genome expression monitoring systems. The transcriptome analysis in *E. coli* indicates that more than 400 genes or ~10% genes in genome are affected in the absence of Lrp, of which at least 130 are suggested to be under the direct control of Lrp (Cho et al., 2008). Previous acetylation proteome identified that Lrp is an acetylated protein (Baeza et al., 2014), and the lysine 36 (K36) is an acetylation site located in its amino-proximal helix-turn-helix DNA-binding domain (Hart et al., 2011). In this work, we further validated that K36Q [substituted by glutamine (Q), mimicking acetylation] can not be acetylated by Pat *in vitro*. Acetylation of K36 inhibits DNA-binding ability of Lrp, and further alters the transcription of *lrp* and *lrp*-regulated genes. In addition, the acetylation of Lrp impairs the type 1 fimbriae production. In conclusion, we demonstrate that (de)acetylation of Lrp is regulated by Pat and CobB, and the reversible acetylation of Lrp modulates its DNA-binding ability, changing the fimbriae production. These findings provide a new example that the acetylation modification of transcription factors is a conserved regulatory fashion of gene expression shared in eukaryotes and bacteria.

## Materials and Methods

### Bacterial Strains, Plasmids, Primers, and Media

All strains, plasmids and primers used in this study were described in Supplementary Tables S1 and S2. Luria-Bertani broth (LB) (per liter, 5 g NaCl, 5 g yeast extract and 10 g tryptone) was used in this work, agar plates contained 1.5% (W/V) agar and supplemented with antibiotics as required. The antibiotics used were 100 μg/ml of ampicillin, 17 μg/ml of chloramphenicol, 50 μg/ml of kanamycin and 50 μg/ml of spectinomycin.

### Generation of *Salmonella* Typhimurium Two-Hybrid Genome Fragment Libraries

The protocol to construct genome fragment libraries was described previously with some modification (Houot et al., 2012). The genomic DNA of *S.* Typhimurium strain 14028s was prepared using PureLink Genomic DNA kit (Invitrogen) and partially digested by *Sau*3AI. The randomly digested DNA was separated on a 0.8% agarose gel, and fragments ranging in size from 2,000 to 4,000 bp were gel-purified using Qiagen gel extraction kit (Qiagen). Three independent two-hybrid libraries were constructed. The first libraries were generated using the pKT25 vectors (Karimova et al., 1998). The other two libraries used pKT25-derivative vectors in which the polylinker site was shifted to +1 and +2 nt, respectively (Handford et al., 2009). The vectors pKT25, pKT25+1 and pKT25+2 were digested by *BamH* I (New England Biolabs) at 37°C for 2.5 h and dephosphorylated with Shrimp Alkaline Phosphatase (New England Biolabs) at 37°C for 1 h. Several independent pools of DNA fragments were ligated overnight at 16°C into the different pKT25 linearized vectors using T4 ligase (New England Biolabs). The resulting ligation mixture was transformed into *E. coli* DH5α competent cells, and plated on LB-ampicillin plates. The resulting transformants were collected by scraping the plates with a glass spreader, and pooled before plasmid isolation. The quantity of each final plasmid library was evaluated under UV illumination.

### Bacterial Two-Hybrid Assay

The protein–protein interaction was detected by Bacterial Adenylate Cyclase Two-Hybrid System Kit (Euromedex) according to the product manuals.

Libraries were tested independently against each pUT18 bait (pKT25-SalLib for *pat* or *cobB*, pKT25+1-SalLib for *pat* or *cobB* and pKT25+2-SalLib for *pat* or *cobB*). Basically, 25–50 ng of each p KT25-derivative library was transformed into 100 μl of electrocompetent BTH101 cells carrying the pUT18 bait vector and plated on screening medium containing 5-bromo-4-chloro-3-indolyl- β-D-galactopyranoside (40 μg/ml, X-gal) and isopropyl-β-D-thiogalactopyranoside (0.5 mM, IPTG). Bacteria expressing interacting hybrid proteins will form blue colonies on LB/X-Gal medium, while cells expressing non-interaction proteins will remain white. IPTG was used to increase β-galactosidase expression. A co-transformant containing pKT25-zip and pUT18-zip was used as a positive control for expected growth on the screening medium. A co-transformant containing empty vector pKT25 and pUT18 was used as a negative control. The blue colonies were picked up and recultivated in liquid medium, and plasmids were isolated and further analyzed by DNA sequencing.

To further validate the results from high-throughput two-hybrid assays, the detected genes (*rhaR*, STM14_1074, *lrp*) and *pat* or *cobB* genes were cloned into pKT25 and pUT18, respectively. The BTH101 strain was co-transformed with the recombinant vectors pKT25-*rhaR*, pKT25-STM14_1074, pKT25-*lrp* and pUT18-*pat* or pUT18-*cobB*, and then spotted onto screening medium.

### Generation of Chromosome Knock-in Strains

Fusion PCR and λ Red recombination were employed to construct *S.* Typhimurium Lrp K36Q, K36R, K36A and the wild type strain. The chloramphenicol resistance cassette was inserted between *lrp* and *ftsK*. PCR products were gel extracted and electroporated into *S.* Typhimurium containing pKD46 prepared in the presence of 10 mM arabinose. The knock-in strains were verified by PCR and DNA sequencing.

### Site-Directed Mutagenesis of *lrp*

Site-directed mutagenesis of *lrp* was performed with the corresponding primers (Supplementary Table S2), using KOD-Plus-Mutagenesis Kit according to the manufacturer’s recommendations (Toyobo). The resulting gene mutations were confirmed by DNA sequencing.

### Expression and Purification of His-Tagged Lrp, Lrp Derivative Mutants, RhaR, STM14_1074, NrdF, FliT, Pat, and CobB

For purification of Lrp and its derivative mutants, pET22b-*lrp*, pET22b-*lrp* K36Q, pET22b-*lrp* K36R, and pET22b-*lrp* K25Q were constructed and verified by sequencing. All the constructed plasmids were transformed individually into *E. coli* strain BL21, and the resultant strains were grown in LB medium containing 100 μg/ml ampicillin at 37°C. IPTG was added to a final concentration of 0.5 mM at the time point when the absorbance of the culture at OD600 ~0.8. The culture was continuously incubated at 37°C for 2 h. Afterward cells were harvested by centrifugation and stored at -80°C. All subsequent procedures were performed at 4°C. Thawed bacteria were resuspended in lysis buffer [50 mM Tris–HCl, 0.5 M NaCl, 10% (V/V) glycerol, pH 7.6], and were broken by high pressure cracker. Any insoluble material was removed by centrifugation for 60 min at 19,000 *g*. The soluble extract was applied to a 1 ml column of nickel-nitrilotriacetic acid (Ni-NTA) agarose (Qiagen) that had been equilibrated with lysis buffer containing. The column was subsequently washed with 10 ml of buffer (50 mM Tris–HCl, 0.5 M NaCl, 10% (V/V) glycerol, 20 mM imidazole pH 7.6), and eluted stepwise with 2 ml aliquots of buffer containing 500 mM imidazole. For the protein whose purity was less than 90%, a further purification step was applied in a 1 ml HiTrap Q HP anion-exchange columns (GE Healthcare) as described previously (Bachhawat and Stock, 2007). The polypeptide compositions of the column fractions were monitored in 15% SDS–PAGE, and subsequent staining with Coomassie bright blue.

For expression of site-specifically acetylated Lrp (K36Ac). *E. coli* strain BL21 was transformed with plasmid pAcKRS-3 and pCDF-PylT-*lrp* K36 (TAG) and then were grown overnight in LB supplemented with 50 μg/ml kanamycin and 100 μg/ml spectinomycin (LB-KS). One liter of prewarmed LB-KS was inoculated with 20 ml overnight culture and was incubated at 37°C. When OD600 reached 1.5, the culture was supplemented with one liter of fresh LB with 20 mM acetyllysine (AcK). Protein expression was induced by addition of 0.5 mM IPTG for 6 h at 37°C, cells were harvested by centrifugation, and stored at -80°C. The purification method was same as Lrp.

For purification of RhaR, STM14_1074, NrdF and FliT, the plasmids pET22b-*rhaR*, pET22b-STM14_1074, pET22b-*nrdF*, pET22b-*fliT* were constructed and verified by DNA sequencing. Expression and purification of RhaR, STM14_1074, NrdF and FliT were performed as Lrp. The protocols to purify Pat and CobB were described previously (Ren et al., 2016).

### *In vitro* Acetylation Assay

The Pat-mediated *in vitro* acetylation reaction was performed as described previously (Ren et al., 2016). The *in vitro* acetylation reaction buffer containing 50 mM Tris-HCl (pH 8.0), 0.1 mM EDTA, 10% glycerol, 1 mM dithiothreitol and 10 mM sodium butyrate. The acetylation was carried out by adding 10 μg Pat, 12.5 mM acetyl-CoA, in a volume of 50 μl. Reaction mixtures were completely mixed and incubated at 37°C for 3 h. Specific protein concentrations are indicated in figure legends.

The AcP-mediated *in vitro* acetylation reaction in the buffer system containing 20 mg/ml bovine serum albumin (BSA), 50 mM Tris-HCl (pH 7.5), 150 mM NaCl, 1 mM EDTA. Lrp was mixed with freshly prepared AcP (20 mM, potassium lithium salt, Sigma) at 37°C for 2 h.

### *In vitro* Deacetylation Assay

The CobB-mediated *in vitro* deacetylation reaction was performed as described previously (Ren et al., 2016). Deacetylation reaction by CobB was performed in deacetylation reaction buffer including 50 mM Tris-HCl (pH 8.0), 135 mM NaCl, 2.5 mM KCl, 1 mM MgCl_2_ in the presence or absence of 1 mM NAD^+^, and in the presence or absence of 10 mM nicotinamide (NAM), an inhibitor for CobB (Bitterman et al., 2002). Specific protein concentrations and reaction times are indicated in figure legends.

### Protein Quantification

Protein concentration was determined using the Bradford reagent with BSA as a standard (Bradford, 1976).

### Western Blot

The standard Western blot procedure was described previously (Ren et al., 2016). For acetylation Western blot, 50 mM Tris (pH 7.5) with 10% (v/v) Tween-20 and 1% peptone was used for blocking and 50 mM Tris (pH 7.5) with 0.1% peptone was used to prepare primary and secondary antibodies.

### Electrophoretic Mobility Shift Assay (EMSA)

DNA fragments used for EMSA were amplified by PCR using *Salmonella* Typhimurium strain 14028s genomic DNA as a template. The promoter region of *fimZ* was amplified using primers *fimZ*-F (5′FAM-ACACAGTGGAGCAATAATAA) and *fimZ*-R (5′FAM-CAGAATATCCTACCCGCTAT). PCR amplification rendered fragments of 202 bp. Approximately 1 nmol of 5′FAM-labeled DNA in a 20 μl volume was incubated at 25°C for 20 min with the indicated amounts of purified the wild type Lrp or mutants. The binding buffer used for protein-DNA incubations contained 100 mM Tris-HCl (pH 8), 375 mM NaCl, 25 mM MgCl_2_, 5 mM DTT, 65% glycerol. Samples were separated on a 5% non-denaturing Tris-glycine polyacrylamide gel at 4°C. FAM-labeled fluorescence was detected by the Fujifilm FLA7000.

### Quantitative Real-Time PCR Assay

Bacteria were cultured in LB broth to log phase, harvested and disrupted by Bead beaters. RNA was isolated using Trizol (Invitrogen), and DNase I digestion was conducted as described previously (Sang et al., 2016). Primers for qPCR were listed in Supplementary Table S2. Samples were run in triplicates and amplified using SYBR Premix Ex Taq II (TAKARA). The relative transcriptional level was determined by the methods of 2^-ΔΔCt^ (Augagneur et al., 2007). 16S rRNA was used as a reference gene.

### Agglutination Assay

The yeast agglutination assay was carried out according to the previous protocol with some modification (Roe et al., 2001). A single colony was used to inoculate 5 ml LB broth, cultured at 37°C without shaking, then subcultured twice for overnight static growth. Agglutination was assayed in slide by mixing 5 μl bacterial culture with an equal volume of bakers’ yeast suspension (10 mg/ml). Mannose inhibition of agglutination was confirmed using 3% α-D-mannose in the yeast suspension.

## Results

### Screening the Partners of Pat and CobB by Bacterial Two-Hybrid System

To screen the protein targets of Pat or CobB, the bacterial two-hybrid system (BTH) was employed. At first, the *S.* Typhimurium strain 14028s genome libraries were constructed. The first one, designated pKT25-SalLib and in order to get a large coverage of the *S.* Typhimurium genome, we engineered two additional libraries (pKT25+1-SalLib and pKT25+2-SalLib) in which genome libraries were cloned in the pKT25 vector or shifted to +1 and +2 nt in the pKT25+1 and pKT25+2 vectors, respectively. Thus, the final *S.* Typhimurium prey libraries consisted of 33000 clones, and were estimated to represent 4.39-fold coverage of *S.* Typhimurium genome.

Next, acetyltransferase Pat and deacetylase CobB proteins were used to test our high-throughput *S.* Typhimurium two-hybrid library with the aim of identifying unknown targets. At last, in total of 12 DNA fragments encoded proteins were screened and confirmed by DNA sequencing (Supplementary Table S3), and eight proteins interacted with Pat, while four proteins with CobB. Notably, four proteins including STM14_1074, RhaR, FliT and Lrp are transcriptional regulators, suggesting that acetylation is an important regulation mechanism for the activity of regulators in bacteria (Thao et al., 2010; Hu et al., 2013; Ren et al., 2016).

**FIGURE 1:**
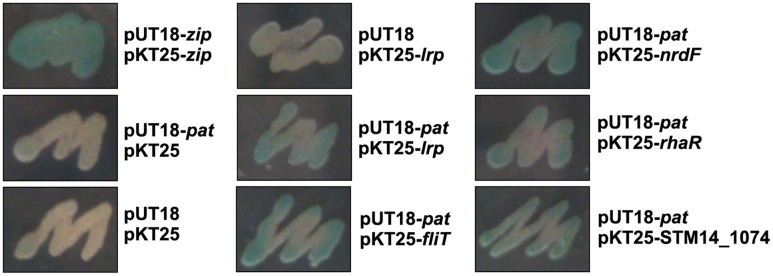
**The bacterial two-hybrid assay.** Interactions between Pat and several indicated proteins were tested on screening medium. The blue colonies represented the detected proteins which had interaction with Pat, while the white colonies had no-interaction with Pat. Co-transformant containing pKT25-zip and pUT18-zip as a positive control. Co-transformant containing pKT25 and pUT18 as a negative control. Each unit represented the corresponding co-transformant in the plates was indicated in Figure.

To confirm the above BTH results, we selected several positive clones, which were encoding regulators (*nrdF*, *rhaR*, *STM14_1074*, *fliT*, *lrp*) and cloned their full-length genes into pKT25 individually. Since bacteria expressing interacting hybrid proteins formed blue colonies on LB/X-Gal medium, as shown in **Figure 1**, each blue co-transformant represented expressing Pat and protein interacted with Pat, suggesting these candidate proteins may exist acetylation modification.

### Validation of Selected Protein by the (de)acetylation Modification *In vitro*

**FIGURE 2:**
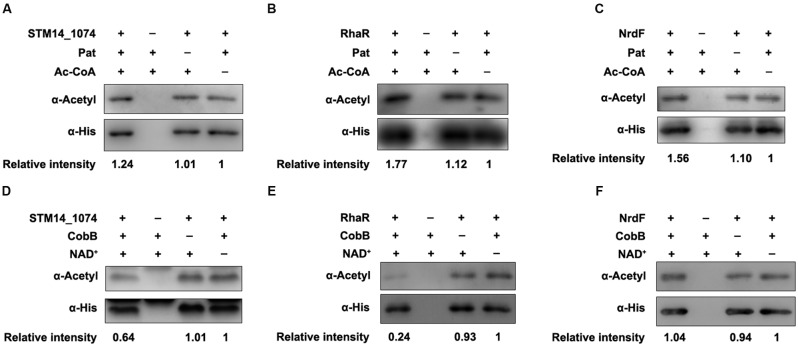
**The acetylation and deacetylation modification of STM14-1074, RhaR and NrdF *in vitro*. (A–C)** Pat acetylates STM14-1074, RhaR and NrdF *in vitro*. Purified STM14-1074, RhaR and NrdF (0.2 μg/μl) incubated with or without Pat (0.2 μg/μl) and Ac-CoA (0.2 mM). **(D–F)** CobB deacetylates STM14-1074, RhaR and NrdF *in vitro*. STM14-1074, RhaR and NrdF (0.2 μg/μl) was purified and incubated with or without CobB (0.1 μg/μl), NAD^+^ (1 mM). The acetylation levels were determined by Western blot. Western blots are representative of at least three independent replicates.

**FIGURE 3:**
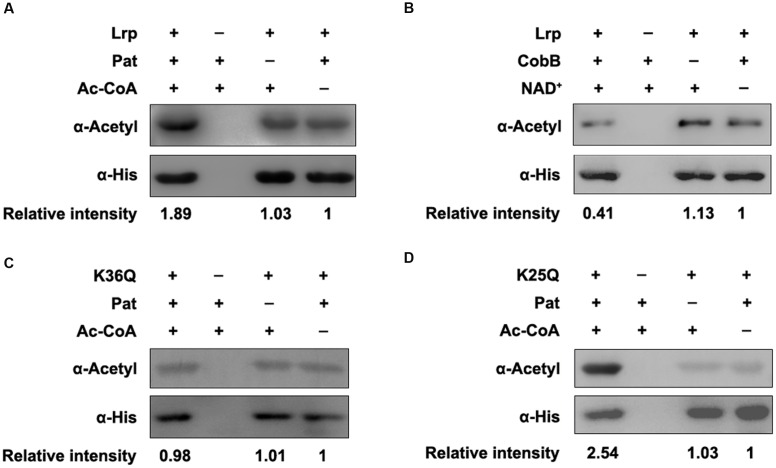
**K36 of Lrp is the acetylation site by Pat. (A)** Pat acetylates Lrp *in vitro*. Purified Lrp (0.2 μg/μl) incubated with or without Pat (0.2 μg/μl) and Ac-CoA (0.2 mM) in a volume of 50 μl. Reaction mixtures were completely mixed and incubated at 37°C for 3 h. The acetylation levels were determined by Western blot. **(B)** CobB deacetylates Lrp *in vitro*. Lrp (0.2 μg/μl) was purified and incubated with or without CobB (0.1 μg/μl), NAD^+^ (1 mM) at 25°C for 6 h. The acetylation levels were determined by Western blot. **(C)** Pat acetylates K36Q *in vitro*. **(D)** Pat acetylates K25Q *in vitro*. Western blots are representative of at least three independent replicates.

To further validate these regulators can be (de)acetylated, the (de)acetylation modification assays *in vitro* were performed. The purified proteins were incubated with Pat or CobB, respectively, and the acetylation levels of each protein were evaluated by the pan anti-acetyllysine antibody (α-Acetyl). After Pat treatment at 37°C for 3 h, the acetylation levels of STM14_1074 (**Figure 2A**), RhaR (**Figure 2B**), NrdF (**Figure 2C**), and Lrp (**Figure 3A**) increased 1.24, 1.77, 1.56, and 1.89-fold, respectively, indicating that NrdF, STM14_1074, RhaR and Lrp can be acetylated by Pat *in vitro*. However, no acetylation signal increase was detected in FliT after incubation with Pat (Supplementary Figure S1).

Then, the CobB-mediated *in vitro* deacetylation reaction was performed at 25°C for 6 h, afterward, the samples were applied to detect the acetylation levels. The acetylation levels of STM14_1074 (**Figure 2D**), RhaR (**Figure 2E**), and Lrp (**Figure 3B**) decreased after CobB incubation, indicating these three proteins can be deacetylated by CobB. However, there was no decrease in the acetylation level of NrdF with CobB treatment (**Figure 2F**).

### Lysine 36 of Lrp Is the Acetylation Site Modified by Pat

Although the role of acetylation in Lrp activity has not been studied, a whole-proteome analysis uncovers that Lrp can be acetylated in *E. coli*, and lysine 25 (K25) and lysine 36 (K36) are the acetylated sites (Baeza et al., 2014). To validate whether these lysine residues are acetylated by Pat, K25 and K36 were site-directed mutated to glutamine (Q) individually mimicking acetylation status of lysine. The acetylation level of K36Q did not increase after Pat-mediated acetylation (**Figure 3C**). However, the acetylation level of K25Q was elevated with Pat treatment (**Figure 3D**). These results indicate that K36 is a key acetylation site by Pat, rather than K25.

### The Lysine 36 (K36) Is a Key Residue for DNA-Binding Ability of Lrp

**FIGURE 4:**
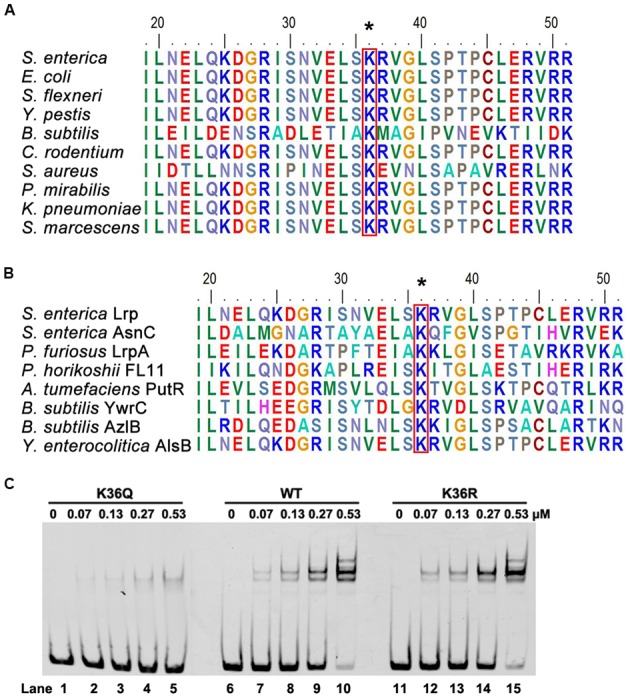
**K36 is essential for the DNA-binding ability of Lrp. (A)** Conservation analysis of Lrp K36 by sequence alignment. Arrows denotes the conserved lysine residues, and the result was analyzed by BioEdit 7.0. **(B)** Conservation analysis of Lrp with other Lrp/AsnC family proteins by sequence alignment. **(C)** DNA-binding abilities of Lrp and derivatives by EMSA. EMSA was used to test the binding of the indicated concentrations of Lrp (lanes 2 to 5, 7 to 10, 12 to 15) to 6′-FAM-labeled *fimZ* promoter. Lanes 1, 6, and 11 represents the labeled DNA alone. From left to right panel: Lrp K36Q, the wild type Lrp, Lrp K36R. EMSA result is representative of at least three independent replicates.

With sequence alignment, we found Lrp K36 is highly conserved in bacteria (**Figure 4A**), and this residue was also conserved in several other DNA binding proteins (**Figure 4B**). In addition, K36 is located in the helix-turn-helix (HTH) DNA-binding domain (Hart et al., 2011). Therefore, we propose that acetylation of K36 may be involved in regulating Lrp activity, specifically the DNA-binding ability of Lrp. To test this hypothesis, K36 was mutated to arginine (R) or glutamine (Q) individually. Whereas the K-to-R substitution avoids acetylation but keeps positive charge, thus mimicking the non-acetylated form, the K-to-Q substitution mimics the constitutively acetylated form through neutralization of the positive charge (Luo et al., 2004). The wild type Lrp, K36Q and K36R proteins were overexpressed and purified to apparent homogeneity (Supplementary Figure S2). Electrophoretic mobility shift assay (EMSA) was performed by incubating the above purified wild type Lrp and its derivatives with 6′-FAM-labeled *fimZ* promoter, which is bound directly by Lrp (McFarland et al., 2008), followed by non-denaturing PAGE analysis. EMSA results showed that both the wild type Lrp and K36R had the similar and strong DNA-binding affinity to the *fimZ* promoter, while K36Q possessed a significantly weaker ability to bind the *fimZ* promoter (**Figure 4C**). These findings indicate that K36 residue is indispensable for DNA-binding ability of Lrp, and suggest that the acetylation of K36 is associated with DNA-binding ability of Lrp. We also determined the DNA-binding ability of K25 by EMSA, since this residue also is acetylated and located in HTH domain. The results showed that mutation of K25 did not affect the DNA-binding ability of Lrp (Supplementary Figure S3).

### Site-Specific Acetylation at K36 Shows Deficiency in DNA-Binding Ability of Lrp

**FIGURE 5:**
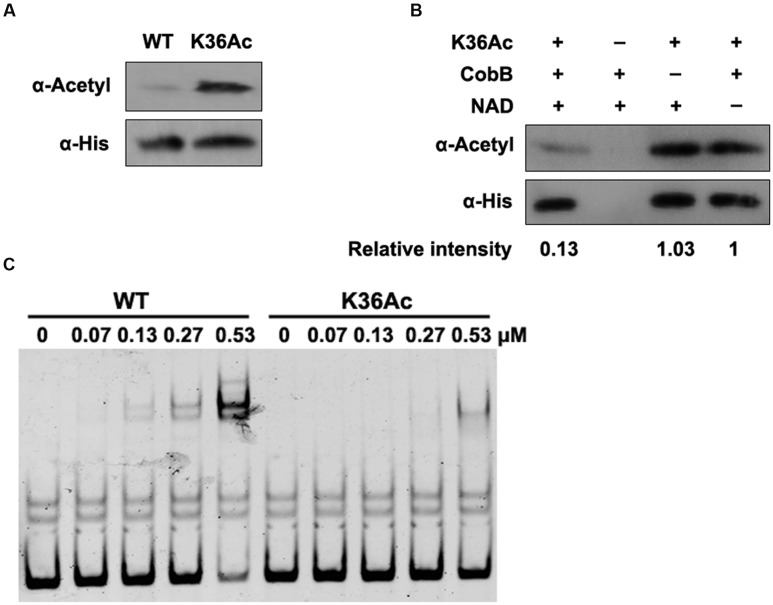
**Acetylation of K36 impairs the binding ability of Lrp to *fimZ* promoter. (A)** The acetylation level of Lrp K36Ac. The wild type Lrp and Lrp K36Ac were purified as described in section “Materials and Methods.” The acetylation levels were determined by Western blot using the pan anti-acetyl lysine antibody. Western blots are representative of two independent replicates. **(B)** CobB deacetylates Lrp K36Ac *in vitro*. Lrp K36Ac (0.2 μg/μl) was incubated with or without CobB (0.1 μg/μl), NAM (10 mM) and NAD^+^ (1 mM). The acetylation levels were determined by Western blot. Western blots are representative of two independent replicates. **(C)** DNA-binding abilities of Lrp and Lrp K36Ac. EMSA was used to test the binding of Lrp at indicated concentrations to 6′-FAM-labeled *fimZ* promoter. Left panel: The wild type Lrp was incubated with probes. Right panel: Lrp K36Ac was incubated with probes. EMSA is representative of at least three independent replicates.

The above EMSA results demonstrate that Lrp K36 is an essential site for DNA-binding affinity, which may be regulated by acetylation. In order to confirm the role of K36 acetylation on activity of Lrp, the site-specific acetylation incorporated N^𝜀^-acetyllysine system was used (Neumann et al., 2008), which has been applied successfully in acetylation of another transcription factor in bacteria (Ren et al., 2016). The *lrp* gene carrying a TAG stop codon at position 36 was co-expressed with an orthogonal N^𝜀^-acetyllysyl-tRNA synthetase/tRNA_CUA_ pair cultured in LB medium supplemented with N^𝜀^-acetyllysine. K36Ac was purified to apparent homogeneity (Supplementary Figure S4), and the incorporation of acetylated lysine of K36Ac was verified by Western blot with pan anti-acetyllysine antibody. The acetylation level of K36Ac increased significantly compared with the wild type Lrp (**Figure 5A**). Moreover, the acetyl groups in K36 could be effectively removed by CobB in an NAD^+^ dependent manner (**Figure 5B**).

Next, K36Ac was used to evaluate the effect of acetylation at K36 on DNA-binding affinity of Lrp by EMSA. The wild type Lrp and K36Ac were incubated with 6′-FAM-labeled *fimZ* promoter followed by EMSA to compare their DNA-binding abilities. Compared with the non-site-specific wild type Lrp, K36Ac possessed a reduced DNA-binding ability (**Figure 5C**), similar as K36Q mimicking acetylation (**Figure 4C**). These results indicate that acetylation of K36 impairs the DNA-binding ability of Lrp.

### Acetylation of K36 Affects the Expression of Lrp Regulon

**FIGURE 6:**
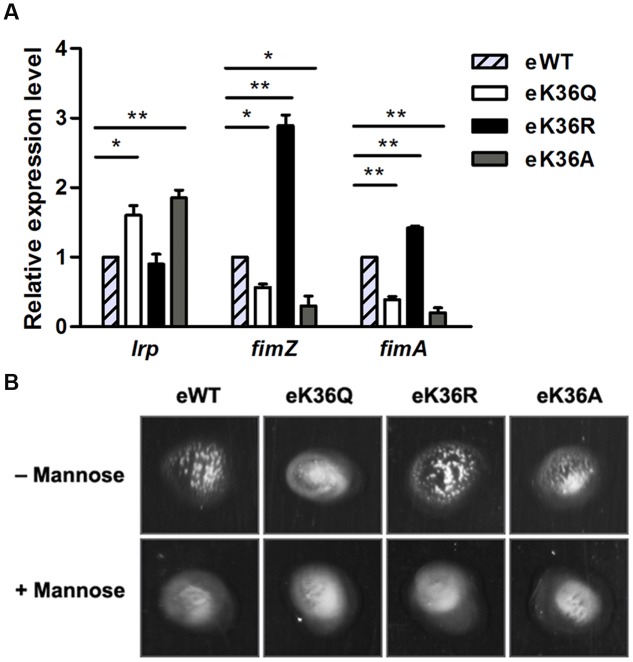
**The effect of acetylation of K36 *in vivo*. (A)** The mRNA levels of Lrp-regulated genes in chromosome *lrp* mutants. Bacteria were grown to log phase, and harvested to isolate total RNA. The transcriptional levels of target genes were determined by qPCR with the methods of 2^-ΔΔCt^. The expression of the tested gene was normalized to that of 16S rRNA and compared to eWT. ^∗^*P* < 0.05, ^∗∗^*P* < 0.01, Student’s *t*-test. **(B)** Mannose-sensitive yeast agglutination assay. 5 μl the chromosome *lrp* mutants culture were mixed with equal volume of bakers’ yeast suspension (10 mg/ml) on slide. Mannose inhibition of agglutination was confirmed using 3% α-D-mannose in the yeast suspension.

To test whether the acetylation of K36 is involved in the regulation of Lrp activity *in vivo*, the chromosome K36Q, K36R, and K36A mutations were constructed individually in *S.* Typhimurium strain 14028s, denoted as eK36Q (engineered K36Q), eK36R (engineered K36R), and eK36A (engineered K36A), respectively. Simultaneously, the similar genetic manipulation was performed in the wild type *lrp* and resulted in eWT (engineered WT) as a control. The above genetic manipulations did not affect bacterial growth and transcription of its downstream genes (Supplementary Figure S5). The *fim* operon of *S*. Typhimurium encodes type 1 fimbriae and can be directly and positively regulated by Lrp (McFarland et al., 2008; Baek et al., 2011). Therefore, *fimZ* and *fimA* were selected as representative genes to determine the effect of acetylation of K36 *in vivo*. The qPCR assay demonstrated that the transcription levels of *fimA* and *fimZ* reduced dramatically in eK36Q and eK36A compared with eWT, while eK36R mimicking non-acetylated lysine residue significantly activated the transcription of these genes (**Figure 6A**). The *lrp* gene in *S*. Typhimurium has been found to be negatively autoregulated (McFarland and Dorman, 2008). As expected, the transcription level of *lrp* increased in eK36Q and eK36A, but relatively low in eWT and eK36R (**Figure 6A**). These results suggest that acetylation of K36 inhibits the DNA-binding of Lrp and thus alters the transcription of target genes of Lrp in *S.* Typhimurium *in vivo*.

The type 1 fimbriae are mannose-sensitive agglutination factors that mediate bacterial adhesion to a broad range of eukaryotic cells by interactions with mannosylated glycoproteins. The expression of the type 1 fimbriae can be evaluated by the yeast agglutination assay (Kukkonen et al., 1993; Baumler et al., 1997). Since Lrp can positively regulate the expression of the type 1 fimbriae (Blomfield et al., 1993; Baek et al., 2011), we used the yeast agglutination assay to confirm the role of acetylation in regulating activity of Lrp. The chromosome K36Q, K36R, K36A mutants along with the wild type strain were subcultured twice for overnight static growth, respectively. Agglutination was assayed in slide by mixing 5 μl bacterial culture with an equal volume of 10 mg/ml bakers’ yeast suspension. The results showed that eWT and eK36R caused mannose sensitive yeast agglutination, however, both eK36Q and eK36A failed to induce yeast agglutination (**Figure 6B**). These findings demonstrate that acetylation of Lrp K36 inhibits the production of the type 1 fimbriae by decreasing the binding ability of Lrp with the *fim* operon promoter.

## Discussion

The proteomic studies have identified several acetylated transcription factors in bacteria (Wang et al., 2010; Weinert et al., 2013; Zhang et al., 2013; Kuhn et al., 2014; Liu et al., 2014; Kosono et al., 2015; Schilling et al., 2015). There are several supporting experimental evidences for regulators regulated by acetylation and thus affecting their regulon, such as transcriptional regulator RcsB (Thao et al., 2010; Hu et al., 2013), the two-component system regulator PhoP (Ren et al., 2016). In this study, four transcription regulators were identified as substrates of Pat. Therefore, it is reasonable to consider that acetylation, as conserved regulation mechanism, is employed by regulators to promptly and finely regulate gene expression. Through bacterial two-hybrid method, we also expect to identify more low copy proteins and proteins not only in the cytosol but in the membrane (Houot et al., 2012), since these proteins can be hardly identified by MS due to the preparation of sample during which will remove major membrane proteins by centrifuging. At last, membrane protein YcgO (Na^+^/H^+^ exchangers) and YbjZ (ATP-binding transport protein) were identified in our work. It indicates that bacterial two-hybrid is an important supplement to identify acetylation proteins.

We focused our attention on a conserved GR Lrp screened from bacterial two-hybrid system, and demonstrated that it could be enzymically acetylated by acetyltransferase Pat and deacetylated by NAD^+^-dependent deacetylase CobB *in vitro.* K36 is the acetylation site identified by previous study (Baeza et al., 2014), however, whether acetylation of this site can affect the function of Lrp is unknown. We validated that K36 was the acetylation site by Pat, and acetylation of K36 impaired its DNA-binding ability, while deacetylation of K36 can effectively bind to DNA. Consideration of the positive charge of K36, the molecular mechanism behind the observed attenuated DNA-binding ability of acetylated Lrp at K36 likely results from neutralization of its positive charge. It would disrupt or inhibit the formation of salt bridge with the negatively charged phosphate backbone of DNA in promoter (Arbely et al., 2011). We also constructed the K36 mutation in chromosome to detect the effect of acetylation of K36 *in vivo*, and demonstrated that acetylation of K36 inhibited the Lrp activity as transcription factor. Therefore, both *in vivo* and *in vitro* findings demonstrate that the acetylation of K36 in its HTH domain crippled the DNA-binding activity of Lrp. K25 is also identified as acetylation site by the previous proteomic study (Baeza et al., 2014). K25Q mutation possessed a similar DNA-binding ability to the wild type Lrp, although K25 is also located HTH domain of Lrp. It suggests that the acetylation of K25 is not involved in regulating DNA-binding. Moreover, K36 of Lrp is more conserved than K25 among different bacteria and other DNA-binding proteins containing AsnC-type HTH domain (Willins et al., 1991). Consider that AsnC/Lrp-like proteins are present in both bacteria and archaea (Kyrpides and Ouzounis, 1995) and K36 site is highly conserved, we speculate that the acetylation of this conserved site is likely a general way to modulate the activity of transcription regulators of the AsnC/Lrp family. In contrast, although MS showed Lrp K25 has acetylation modification, the physiological significance of acetylation of K25 need to be further explored.

As a global gene regulator, Lrp regulates the expression of a large number of genes including virulence-related genes in *E. coli* (Jakel et al., 2001; Hung et al., 2002) and *Salmonella* (Wang et al., 1993; Friedberg et al., 1995; Hecht et al., 1996; McFarland et al., 2008). Among them, *fimZ* and *fimA* are shown to be activated by Lrp and consequently allow *S.* Typhimurium to produce type 1 fimbriae (McFarland et al., 2008). In our study, we demonstrate that the acetylated Lrp failed to activate *fimZ* and *fimA* due to its decreased DNA-binding ability, reducing fimbriae production. Although fimbriae facilitate bacterial adherence to epithelial cells and subsequently pathogenesis in host cells (Althouse et al., 2003), their presence on outer membranes may also act as an antigen, which may be a target for the host immune system (Duguid and Campbell, 1969). Therefore, it is critical to switch between antigen-on and antigen-off phases to evade immune attacks. Maybe acetylation of Lrp is a prompt and effective way for *Salmonella* to regulate this switch. Similarly, several other transcription factors are also identified as acetylation proteins, and acetylation inhibits their DNA-binding activities, such as RcsB (Thao et al., 2010; Hu et al., 2013), PhoP (Ren et al., 2016), suggesting that reversible lysine acetylation in the DNA-binding domain is a conserved and novel regulatory mechanism of gene expression across microorganisms.

Protein acetylation in an AcP-dependent manner provides compelling evidence for a recently reported mechanism of bacterial N^𝜀^-lysine acetylation. Compared with Pat-dependent acetylation, the effect of non-enzymatic acetylation of proteins by AcP appears to be quite extensive (Weinert et al., 2013; Kuhn et al., 2014). However, bacterial two-hybrid system can not screen the AcP-dependent acetylation proteins compared with MS method. We further validated that NrdF, STM14_1074 could be acetylated by AcP *in vitro* (Supplementary Figure S6). These results suggest that for one protein, its acetylation can be mediated simultaneously by Pat- and AcP-dependent manners. However, how acetylation from the two manners coordinately regulates the activity of protein is an interesting question for further study.

## Author Contributions

Conceived and designed the experiments: Y-FY and RQ. Performed the experiments: RQ, YS, JR, QZ, and SL. Analyzed the data: RQ, YS, JR, and ZC. Contributed reagents/materials/analysis tools: RQ and QZ. Wrote the paper: YS, JR, ZC, and Y-FY.

## Conflict of Interest Statement

The authors declare that the research was conducted in the absence of any commercial or financial relationships that could be construed as a potential conflict of interest.
