# Supplementary material for: The Bacterial Two-Hybrid System Uncovers the Involvement of Acetylation in Regulating of Lrp Activity in Salmonella Typhimurium
Source: Front Microbiol. 2016 Nov 17;7:1864. doi: 10.3389/fmicb.2016.01864 (PMC5112231; doi:10.3389/fmicb.2016.01864)
Supplement: Supplementary file 1 [file Data_Sheet_1.docx]

**Supplementary Information**

**Supplementary Tables**

**Table S1. Strains and plasmids used in this study**

| **Strains and plasmids** | **Genotypes and characteristics** | **Sources or references** |
| --- | --- | --- |
| **Strains** |  |  |
| *E. coli* DH5α | F- 80*lacZ* M15 (lacZYA–argF)U169*eoRrecA1endA1 hsdR17 phoA supE*44-thi-1 *gyrA96 relA1* | [Ren et al., 2016](#_ENREF_1) |
| *E. coli* BL21 | F- *ompT gal dcm lon hsdSB*(rB- mB-) λ(DE3 [*lacI lacUV*5-T7 gene 1 *ind1 sam7 nin5*]) | Laboratory stock |
| *S.* Typhimurium 14028s | Wild type *S.* Typhimurium strain | [Ren et al., 2016](#_ENREF_1) |
| BTH101 | Non-reverting adenylate cyclase deficient (cya) *E. coli* reporter strain for bacterial two-hybrid system | Euromedex |
| Lrp (eK36Q) | *S.* Typhimurium 14028s Lrp (K36Q) chromosome mutation | This study |
| Lrp (eK36R) | *S.* Typhimurium 14028s Lrp (K36R) chromosome mutation | This study |
| Lrp (eK36A) | *S.* Typhimurium 14028s Lrp (K36A) chromosome mutation | This study |
| Lrp (eWT) | *S.* Typhimurium 14028s Lrp (K36) chromosome genetic manipulation | This study |
| **Plasmids** |  |  |
| pKD46 | Express λ red recombinase | [Ren et al., 2016](#_ENREF_1) |
| pKD3 | Source for chloramphenicol resistance cassette (cat) | [Ren et al., 2016](#_ENREF_1) |
| pCP20 | FLP recombinase | [Ren et al., 2016](#_ENREF_1) |
| pUT18 | Vector for Bacterial two-hybrid system | Euromedex |
| pUT18-*pat* | Amp^R^, pUT18 harboring *pat* | This study |
| pUT18-*cobB* | Amp^R^, pUT18 harboring *cobB* | This study |
| pKT25 | Kan^R^, vector for Bacterial two-hybrid system | Euromedex |
| pKT25+1 | Kan^R^, pKT25-derivative vectors in which the polylinker site was shifted to +1 nt | This study |
| pKT25+2 | Kan^R^, pKT25-derivative vectors in which the polylinker site was shifted to +2 nt | This study |
| pKT25-SalLib | Kan^R^, pKT25 harboring *Salmonella* library | This study |
| pKT25+1-SalLib | Kan^R^, pKT25+1 harboring *Salmonella* library | This study |
| pKT25+2-SalLib | Kan^R^, pKT25+2 harboring *Salmonella* library | This study |
| pUT18-zip | Amp^R^, Bacterial two-hybrid system control plasmid | Euromedex |
| pKT25-zip | Kan^R^, Bacterial two-hybrid system control plasmid | Euromedex |
| pKT25*-rhaR* | Kan^R^, pKT25 harboring *rhaR* | This study |
| pKT25-STM14_1074 | Kan^R^, pKT25 harboring STM14_1074 | This study |
| pKT25-*fliT* | Kan^R^, pKT25 harboring *fliT* | This study |
| pKT25-*nrdF* | Kan^R^, pKT25 harboring *nrdF* | This study |
| pKT25-*lrp* | Kan^R^, pKT25 harboring *lrp* | This study |
| pQE80-*pat* | Amp^R^, pQE80 harboring *pat* | Laboratory stock |
| pQE80-*cobB* | Amp^R^, pQE80 harboring *cobB* | Laboratory stock |
| pET22b | Expression vector | Laboratory stock |
| pET22b-*rhaR* | Amp^R^, pET22b harboring *rhaR* | This study |
| pET22b-STM14_1074 | Amp^R^, pET22b harboring STM14_1074 | This study |
| pET22b-*fliT* | Amp^R^, pET22b harboring *fliT* | This study |
| pET22b-*nrdF* | Amp^R^, pET22b harboring *nrdF* | This study |
| pET22b-*lrp* | Amp^R^, pET22b harboring *lrp* | This study |
| pET22b-*lrp* (K36Q) | Amp^R^, pET22b harboring *lrp* (K36Q) | This study |
| pET22b-*lrp* (K36R) | Amp^R^, pET22b harboring *lrp* (K36R) | This study |
| pET22b-*lrp* (K25Q) | Amp^R^, pET22b harboring *lrp* (K25Q) | This study |
| pAcKRS-3 | Kan^R^, coding for *M. barkeri*  pyrrolysine tRNA synthetase | Gift from Jason Chin |
| pCDF-*pylT* | Spe^R^, pCDFduet harboring *pylT* | Gift from Jason Chin |
| pCDF*-pylT* -*lrp* | Spe^R^, pCDF PylT harboring *lrp* | This study |
| pCDF*-pylT* -*lrp* (K36TAG) | Spe^R^, pCDF PylT harboring *lrp* (K36TAG) | This study |

**Table S2. Primers used in this study**

| **Primer Name** | **Primer Sequence** |
| --- | --- |
| pKT25-Check-F | gacggcggatatcgacatgt |
| pKT25-Check-R | atgtgctgcaaggcgattaa |
| pKT25+1-F | agggtcgactctagatggatccccgggtacc |
| pKT25+1-R | ggtacccggggatccatctagagtcgaccct |
| pKT25+2-F | agggtcgactctagagaggatccccgggtacc |
| pKT25+2-R | ggtacccggggatcctctctagagtcgaccct |
| pUT18-Check-F | cagctggcacgacaggtttcccg |
| pUT18-Check-R | atacgacgaaaatcttctcatct |
| pUT18-*pat*-*Hind* III-F | cccaagcttg atgagccagcaaggactggaa |
| pUT18-*pat*-*Xba* I-R | gctctagagtcgattcatcacatttggccaga |
| pUT18-*cobB-Xba* l-F | gctctagagatgcagtcgcgtcggtttcat |
| pUT18-*cobB*-*EcoR* I-R | ggaattcgacagccctttcaggaatttatcaa |
| pKT25-*rhaR*-*BamH* I-F | cgggatcccgtggcaaatcagttaatccttt |
| pKT25-*rhaR*-*Kpn* I-R | ggggtaccttaatcgctttgattactgagatg |
| pKT25-STM14_1074-*BamH* I-F | cgggatcccatgataaaaacggatctcaatga |
| pKT25-STM14_1074-*Kpn* I-R | ggggtaccttactcttccaccattcgcg |
| pKT25-*lrp*-*BamH* I-F | cgggatcccatggtagatagcaagaagcg |
| pKT25-*lrp*-*Kpn* I-R | ggggtaccttagcgtgtcttaataaccag |
| pKT25-*narD*-*BamH* I-F | cgggatcccatgaaattatctcgtattagcgc |
| pKT25-*narD*-*Kpn* I-R | ggggtaccttaaaaattccagtcttcgtctt |
| pKT25-*fliT*-*BamH* I-F | cgggatcccatgacctcaaccgtggagttta |
| pKT25-*fliT*-*Kpn* I-R | ggggtaccttatgaggcgccaggcgcat |
| pET22b-Check-F | taatacgactcactataggg |
| pET22b-Check-R | gctagttattgctcagcgg |
| pET22b-*rhaR*-*EcoR* I-F | cggaattcgtggcaaatcagttaatcct |
| pET22b-*rhaR*-*Xho* I-R | cggaattcgtggcaaatcagttaatcct |
| pET22b-STM14_1074-*EcoR* I-F | ccctcgagtgaggcgccaggcgcatctg |
| pET22b-STM14_1074-*Xho* I-R | ccctcgagctcttccaccattcgcgcat |
| pET22b-*lrp*-*EcoR* I-F | cggaattcatggtagatagcaagaagcg |
| pET22b-*lrp*-*Xho* I-R | ccctcgaggcgtgtcttaataaccagac |
| *lrp*-K25Q-F | cttaatgaactgcaacaggatgggcgtatttcc |
| *lrp-*K25Q-R | ggaaatacgcccatcctgttgcagttcattaag |
| *lrp*-K36Q-F | aacgtcgagctttctcaacgagtaggactttcg |
| *lrp-*K36Q-R | cgaaagtcctactcgttgagaaagctcgacgtt |
| *lrp*-K36R-F | aacgtcgagctttctcgacgagtaggactttcg |
| *lrp-*K36R-R | cgaaagtcctactcgtcgagaaagctcgacgtt |
| *lrp*-K36A-F | aacgtcgagctttctgcacgagtaggactttcg |
| *lrp-*K36A-R | cgaaagtcctactcgtgcagaaagctcgacgtt |
| *fimZ*-promoter-F | 5’FAM-acacagtggagcaataataa |
| *fimZ*-promoter-R | 5’FAM-cagaatatcctacccgctat |
| pCDFDuet-*lrp*-*Nco* I-F | ctagccatgggccatcatcatcatcatcatgtagatagcaagaagg |
| pCDFDuet*-lrp*-*Xho* I-R | ccctcgagttagcgtgtcttaataaccagac |
| pCDFDuet*-*Check-F | gaaattaatacgactcacta |
| pCDFDuet*-*Check-R | gtttagaggccccaaggggttat |
| *lrp*-K36-TAG-F | aacgtcgagctttcttagcgagtaggactttcg |
| *lrp-*K36-TAG-R | cgaaagtcctactcgctaagaaagctcgacgtt |
| genome-*lrp*-mutant-F1 | atagcatgaaccgatgaacg |
| genome-*lrp*-mutant-R1 | gggcgcttcttgctatctaccattattattgtctctctgtat |
| genome-*lrp*-mutant-F2 | atacagagagacaataataatggtagatagcaagaagcgccc |
| genome-*lrp*-mutant-R2 | gaagcagctccagcctacacttaatgatgatgatgatgatggcgtgtcttaataaccagac |
| genome-*lrp-*mutant-F3 | catcatcatcatcatcattaagtgtaggctggagctgcttc |
| genome-*lrp-*mutant-R3 | gattttgcacctgttccgtgccatatgaatatcctcctta |
| genome-*lrp-*mutant-F4 | taaggaggatattcatatggcacggaacaggtgcaaaatc |
| genome-*lrp-*muant-R4 | gcaggaagtgagaatcagcg |
| genome-*lrp*-Check-F | attgtagggaatttacagac |
| genome-*lrp-*Check-R | cggcagcccaaatacagaga |
| 16s-qPCR-F | cagccacactggaactgaga |
| 16s-qPCR-R | gtgcttcttctgcgggtaac |
| *lrp*-qPCR-F | tggcaaagatctcgaccgta |
| *lrp*-qPCR-R | ggttcaacagcgccgtatag |
| *fimZ*-qPCR-F | aggccgaagtcaaactggta |
| *fimZ*-qPCR-R | gctcttattgctcttccggc |
| *fimA*-qPCR-F | ataccgtaccgccagcttta |
| *fimA*-qPCR-R | agaggagacagccagcaaat |

**Table S3. The positive clones screened by bacterial two-hybrid system.**

| **Number** | **Bait** | **Prey Proteins** |
| --- | --- | --- |
| 1 | Pat | PrfC (peptide chain release factor 3) |
| 2 | Pat | Lrp (leucine-responsive global transcriptional regulator ) |
| 3 | Pat | NrdF (ribonucleoside-diphosphate reductase 2, beta subunit) |
| 4 | Pat | RhaR (transcriptional activator of rhaSR) |
| 5 | Pat | YbeS (DnaJ family molecular chaperone) |
| 6 | Pat | FliT (flagellar protein) |
| 7 | Pat | STM14_1074 (putative transcriptional regulator) |
| 8 | Pat | Dgt (deoxyguanosine triphosphate triphosphohydrolase) |
| 9 | CobB | IlvA (threonine dehydratase) |
| 10 | CobB | STM14_2960 (putative reductase) |
| 11 | CobB | YbjZ (ABC transporter permease protein) |
| 12 | CobB | YcgO (K(+)/H(+) antiporter) |

**Supplementary Figures**

**Figure S1**


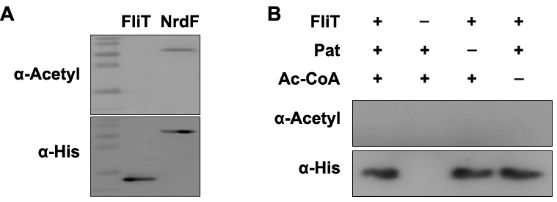


**The acetylation modification FliT *in vitro*.** (A) Acetylation signal of FliT could not be detected. FliT was expressed in *E. coli* strain BL21 and purified as described in *Methods*. The acetylation levels were determined by Western blot. NrdF was used as a positive control. (B) Pat could not acetylate FliT *in vitro*. Purified FliT (0.2 μg/μl) incubated with or without Pat (0.2 μg/μl) at the presence of Ac-CoA (0.2 mM). Western blots are representative of at least three independent replicates.

**Figure S2**

**
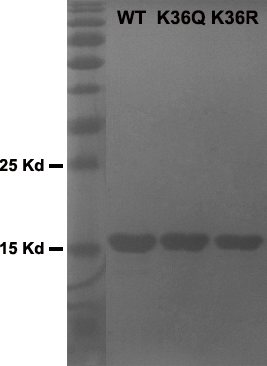
**

**SDS-PAGE analysis of Lrp and K36Q and K36R mutants.** Lrp and its derived mutant proteins were expressed and purified as described in “Materials and Methods”. 1 μg of Lrp and K36Q and K36R were resolved on 15% SDS-PAGE respectively and stained with Coomassie bright blue.

**Figure S3**

**
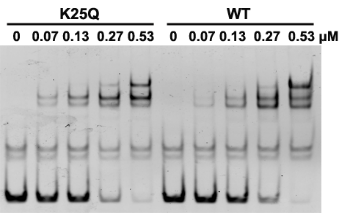
**

**DNA-binding abilities of Lrp and K25Q to *fimZ* promoter.** EMSA was used to test the binding of the indicated concentrations of Lrp and K25Q to 6’-FAM-labeled *fimZ* promoter.

**Figure S4**

**
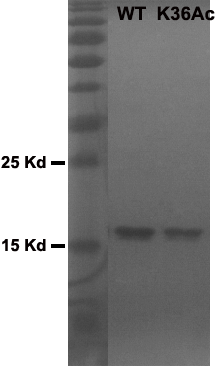
**

**SDS-PAGE analysis of Lrp and K36Ac.** The wild type Lrp (without site-specific acetylation) and K36Ac were expressed and purified as described in “Materials and Methods”. Lrp and K36Ac were resolved on 15% SDS-PAGE and stained with Coomassie bright blue.

**Figure S5**

**
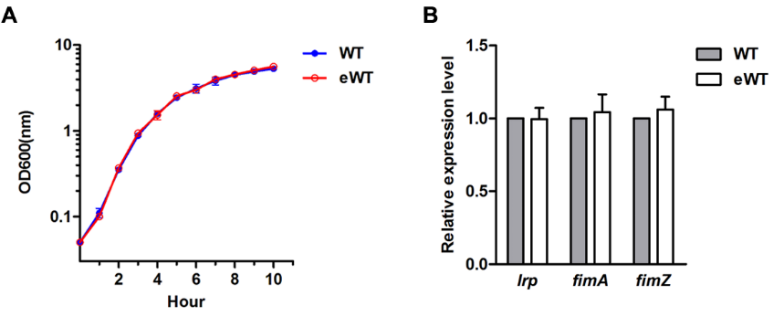
**

**The chromosome genetic manipulation of *lrp* did not affect bacterial growth and transcription of *lrp*, *fimA* or *fimZ*.** We constructed Lrp K36Q, K36R and K36A mutants by replacing the original *lrp* locus in *S.* Typhimurium chromosome. After removing the chloramphenicol resistance cassette at 5’ end of *lrp* locus, there is still a short DNA sequence residual in genome. Lrp eWT (engineered WT) has been performed the same gene manipulation as Lrp K36Q and K36R mutants. Lrp eWT served as the wild type strain when Lrp K36Q, K36R and K36A mutant stains were used. In order to confirm that the DNA sequence residual at 5’ end of *lrp* locus does not affect bacteria growth and transcription of *lrp*, *fimA* or *fimZ*. We compared (**A**) the growth rates and (**B**) *lrp*, *fimA* and *fimZ* mRNA levels between WT and eWT. The construction method was described in “Materials and Methods” section. For growth curve measurement, the strains were cultured overnight, diluted to OD600 ~0.05 with fresh LB medium, and the OD600 was recorded each hour. The cells were grown to log phase (OD600 ~0.4) for RNA isolation and qPCR performance.

**Figure S6**

**
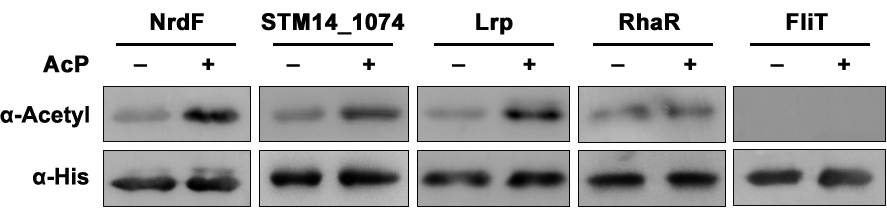
**

**AcP is responsible for acetylation of NrdF and STM14_1074 *in vitro*.** NrdF or STM14_1074 (1 500 ng) was incubated with or without acetyl-phosphate (AcP) (20 mM) at 37°C for 3 h. Products were resolved on 12% SDS-PAGE and probed with anti-His antibody and anti-acetylation antibody.
